# Supplementary material for: Content-rich biological network constructed by mining PubMed abstracts
Source: BMC Bioinformatics. 2004 Oct 8;5:147. doi: 10.1186/1471-2105-5-147 (PMC528731; doi:10.1186/1471-2105-5-147)
Supplement: Additional File 5 — The original Chilibot query results of the term "long-term potentiation (LTP)" and 22 other terms, limiting the latest references analyzed to the years 1990, 1995, 2000, and 2004. [file 1471-2105-5-147-S5.bz2 › chilibotAdditionalFile5/ltp2000/html/LTP_SYNAPTOTAGMIN.html]

 


 **LTP** and **SYNAPTOTAGMIN** 
  
Found 4 abstracts in PubMed,  **4 abstracts were retrieved and analyzed**.  


---

 Search Google  |
 PDF files only 
|  EDU domain only 

---

**Interactive relationship** (e.g. stimulation, inhibition, etc)

**Parallel relationship** (e.g. studied together, co-existance, homology, etc.)

- To explore further the role of the presynaptic terminal in long term potentiation  [ **LTP** ] , we have investigated changes in three synaptic vesicle proteins, synapsin,  **synaptotagmin**  and synaptophysin, in control tissue and in tissue prepared from potentiated dentate gyrus 45 min and 3 h after induction of long term potentiation  [ **LTP** ] .  Ref: 7914357 Neuroscience, 1994
